# Sepiapterin as a treatment for people living with phenylketonuria: a plain language summary of the APHENITY trial

**DOI:** 10.57264/cer-2025-0116

**Published:** 2025-12-09

**Authors:** Ania C Muntau, Sarah Gallagher, Melissa Lah, Amaya Belanger-Quintana, Nicola Longo

**Affiliations:** 1 University Children's Hospital, University Medical Center Hamburg-Eppendorf, Hamburg, Germany; 2 German Center for Child & Adolescent Health, Hamburg, Germany; 3 National PKU Alliance, Roanoke, VA, USA, & patient caregiver; 4 Department of Medical & Molecular Genetics, Indiana University School of Medicine, Indianapolis, IN, USA; 5 Unidad de Enfermedades Metabólicas, Hospital Ramón y Cajal, Madrid, Spain; 6 Department of Human Genetics, University of California, Los Angeles, Los Angeles, CA, USA

**Keywords:** APHENITY, phenylketonuria, plain language summary, sepiapterin

## Abstract

**What is this summary about?:**

This plain language summary is based on an article about the APHENITY trial that was published in *The Lancet* journal in October 2024. The APHENITY trial was a phase 3 clinical trial to find out whether sepiapterin helped people living with phenylketonuria (PKU) and to learn more about its safety. PKU is a rare, genetic condition that causes high levels of phenylalanine (Phe) to build up in the body. High levels of Phe in the body can cause symptoms such as seizures, rashes, and problems with movement, and can also affect brain development, thinking skills, and behaviour. These symptoms can have an impact on people's health-related quality of life.

**What happened in the APHENITY trial?:**

Previous studies have shown that sepiapterin is able to decrease Phe levels in the blood. In the APHENITY trial, the researchers wanted to learn more about the efficacy and safety of sepiapterin in a large group of people living with PKU over the course of 8 weeks of treatment. The researchers wanted to:

- Assess the efficacy of sepiapterin by seeing whether sepiapterin decreased Phe levels in the blood compared with a placebo
- Assess the safety of sepiapterin by seeing how many health complaints the participants who took sepiapterin had compared with those who took the placebo

The APHENITY trial was carried out in two parts:

- During Part 1, the participants took sepiapterin for 2 weeks to find out if it decreased their Phe levels
- During Part 2, the participants who benefitted from sepiapterin during Part 1 were randomly assigned to either continue taking sepiapterin or start taking a placebo for a further 6 weeks

**What were the results?:**

During Part 1, the researchers found that 114 out of 156 participants benefitted from sepiapterin. This was 73% or about 7 in 10 participants.

During Part 2, the researchers found that the participants who took sepiapterin had reduced blood Phe levels compared with those who took the placebo. The researchers also found that compared with those who took the placebo, more of the participants who took sepiapterin reduced their blood Phe levels to within the ranges recommended by treatment guidelines.

For both parts of the trial, the participants did not have any serious health complaints. So, the researchers concluded that no safety concerns were seen with sepiapterin in this trial.

**What do the results of the trial mean?:**

These results showed that sepiapterin helped to reduce blood Phe levels in the participants, and there were no unexpected safety concerns in people living with PKU.

**ClinicalTrials.gov NCT number:** NCT05099640

**This is an abstract of the Plain Language Summary of Publication article.**

To read the full Plain Language Summary of this article, click here to view the PDF.

